# Bicuspid Aortic Valve, from the Unknown till the Perfection of the Species

**DOI:** 10.31083/j.rcm2508310

**Published:** 2024-08-23

**Authors:** Marta Bargagna, Guido Ascione, Edoardo Zancanaro, Francesco Fioravanti, Alessandra Sala, Cinzia Trumello, Guohao Chang, Alessandro Verzini, Alessandro Castiglioni, Francesco Maisano

**Affiliations:** ^1^Department of Cardiac Surgery, IRCCS San Raffaele Hospital, Vita-Salute San-Raffaele University, 20132 Milan, Italy; ^2^Department of Cardiac-Electrophysiology and Arrhythmia, IRCCS San Raffaele Hospital, Vita-Salute San-Raffaele University, 20132 Milan, Italy; ^3^Department of Cardiac Surgery, National University Heart Center, 119074 Singapore, Singapore

**Keywords:** bicuspid aortic valve, aortopathy, aortic valve repair, ascending aorta dilatation, transcatheter aortic valve replacement (TAVR)

## Abstract

The bicuspid aortic valve (BAV) is the most common congenital cardiac abnormality. Though most often isolated, BAV may be associated with other cardiovascular malformations. BAV-related aortopathy is the most common, sharing genetic alterations and phenotypic heterogeneity characteristics. Sometimes silent for a lifetime, BAV may manifest as aortic valve dysfunction, aortic aneurysm, or more emergent situations, such as endocarditis or aortic dissection. Its embryological origin and the characterization of the genes involved, as well as the histopathological and hemodynamic aspects of its natural history, are becoming increasingly clear. In addition, emerging evidence of rhythm disorders associated with BAV has been identified. A new international nomenclature and classification has been introduced to interpret all the advances made in recent years for the comprehension of this condition. In the guidelines, more attention has been paid to the diagnosis of BAV and related aortopathy, together with surveillance, and family screening. Surgical treatment remains the gold standard, especially in young low-risk patients, and valve repair techniques have been shown to be effective and durable. Finally, the new era of transcatheter techniques is also being applied to dysfunctional BAV, allowing the treatment of patients at high surgical risk, with increasingly promising results, and the possibility of expanding indications through the introduction of more advanced devices. This review aims to comprehensively describe the BAV conundrum, focusing on anatomy, pathophysiology, genetics, diagnosis of BAV-related disorders, and the different treatment options available in the transcatheter era.

## 1. Introduction

**Fig. 1.**
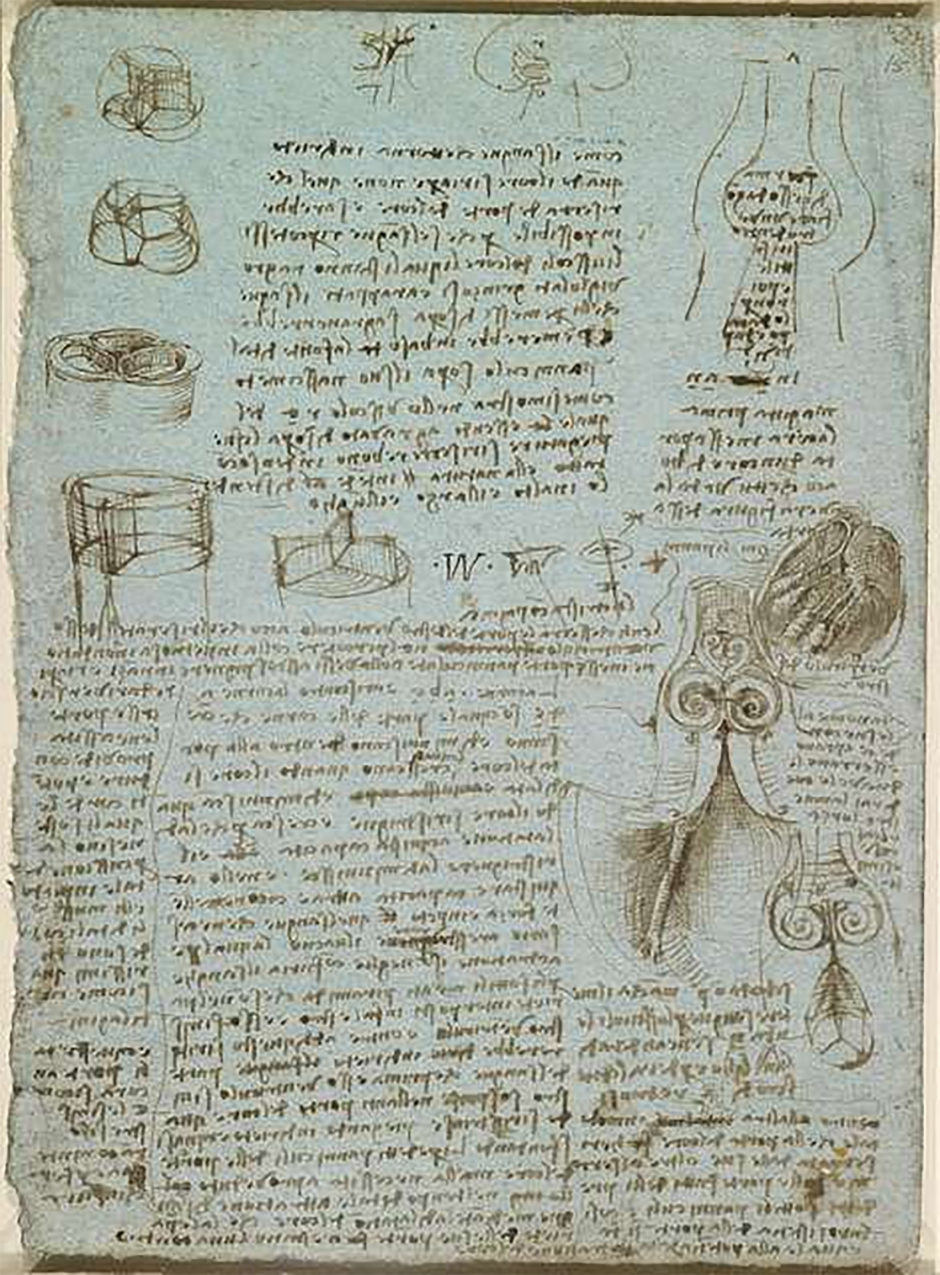
**Leonardo da Vinci, The aortic valve c. 1512-13, Royal Collection of the United Kingdom (public domain picture)**.

First described by Leonardo da Vinci in 1500, bicuspid aortic valve (BAV) is the most common congenital cardiac abnormality, with an estimated 1–2% prevalence in the general population and almost three times higher occurrence in men than in women (Fig. 1) [1, 2, 3, 4]. Although it may remain silent for a whole lifetime, BAV can manifest with heterogeneous symptoms, caused by valve dysfunction, and complications resulting from associated cardiovascular anomalies. In 60–80% of the cases, it is associated with alterations of the aorta, referred to as aortopathies, which increase the risk of aortic aneurysm and dissection and may occur independently from valve dysfunction [5]. Other cardiovascular malformations can be associated with BAV, including coarctation of the aorta and atrial or ventricular septal defects, and up to 18% of BAV can be found in genetic syndromes like Loeys-Dietz, Marfan, and Turner syndrome [6].

This review summarizes the embryological and genetic origins of BAV, its evolution, and complications, up to the most advanced surgical and transcatheter treatment to date.

## 2. Aetiopathogenesis

BAV may either be the result of a new-onset mutation or have an autosomal dominant transmission with sometimes incomplete penetrance and expressivity. A recent meta-analysis, including 23 observational studies, showed that the prevalence of BAV in families with at least one BAV subject was 23.6% and the prevalence of aortic dilatation in BAV patients’ relatives was 29.2% [7]. First-degree relatives of patients with BAV have a 6.88-fold increased risk of a concordant diagnosis if compared with the normal population and, a 3.63-fold higher risk of aortic dissection, which increased to 6.13 in patients with a diagnosis of both BAV and aortic aneurysm [8].

The genetic basis of a BAV is poorly understood given its complex inheritance. Between the 5th and 9th week of embryogenesis, the aortic valve begins to form from the endocardial cushions located at the outflow tract. In this complex process, three different cardiac progenitor cells play a role: the neural crest, second heart field, and endocardial cushion derived cells. The altered regulation of collagen and proteoglycan formation within these structures appears to be a risk factor for the development of BAV and ascending aortopathy, which are closely linked. Grewal *et al*. [9] reviewed and classified some genes and signaling pathways involved in the aortic valve and ascending aortic wall formation (*eNOS*, *NOTCH*, *TGFβ*, *Nkx2.5*, *GATA*, bone morphogenetic protein, vascular endothelial growth factor, *NFATc1*, *Rock 1,2*), and some of them were related to specific BAV subtypes.

Furthermore, less differentiated vascular smooth cells of the great arteries and increased activity of matrix metalloproteinase-9 are frequently found in BAV patients with aortic complications.

Even histopathological, and hemodynamic factors, however, may contribute to the evolution of aortopathies. In the aortic wall of BAV, elastic fiber orientation is altered, fiber mass is reduced in the medial layer and the intimal layer is thinner, thus resulting in a stiffer aortic wall if compared to healthy subjects [10]. The role of shear stress in the mechanism of dilatation of the ascending aorta in BAV patients has often been debated, but, to date, it is considered an additional element with varying degrees of individual impact [10, 11, 12]. Recently Soulat *et al*. [13] evaluated the wall shear stress as a predictor of ascending aorta dilatation in 72 BAV patients (89% of which had none or mild aortic regurgitation), using 4-dimensional flow cardiac magnetic resonance. They identified that larger areas of elevated wall shear stress were associated with a higher rate of aortic dilatation [13]. The Copenhagen Heart Baby Study, in which the authors studied images from transthoracic echocardiograms of 25,556 newborns in Denmark between 2016 and 2018, showed higher flow velocity across the valve and larger aortic root and tubular tract diameter in BAV newborns, with signs of aortopathy in 33.2% of them [2]. There is also evidence that the aortic dilatation rate in BAV is higher than expected for all aortic levels, it is not related to baseline aortic size or BAV type, sex, or blood pressure values and is largely heterogenous, with 43% of BAV not progressing [5, 14].

The multiple mechanisms involved in the etiopathogenesis of this valvulo-aortopathy do not definitively explain the variable clinical manifestations that can be observed in BAV patients [15, 16]. Usually, phenotypic manifestation can involve the valve function, with stenosis, regurgitation, or infective endocarditis. Michelena *et al*. [15] reported that, in people with a normal or mildly dysfunctional BAV during 20 years of follow-up, 24% required aortic valve surgery, 5% ascending aorta surgery, and 27% any cardiovascular surgery. Recently Yang *et al*. [17] analyzed the incidence of morbidity and mortality, during a follow-up of 19.1 years, of 652 BAV patients [median age 37 (22–53) years; 81% adult and 19% pediatric]. They found that the cumulative lifetime risk of ≥ moderate aortic stenosis or regurgitation was 80.3%, aortic valve surgery was 68.5%, aortic aneurysm ≥45 mm (or z-score ≥3) was 75.4%, aorta surgery was 27%, infective endocarditis was 6%, and aortic dissection was 1.6% [17]. BAV stenosis is the main cause of aortic valve replacement in this cohort (occurring in 61–80% of BAV patients), while BAV regurgitation requires surgery in a smaller percentage of patients (15–29%), mostly in those who are younger [18]. Patients with BAV, according to available metanalysis, experience a 12-fold increased risk of valve endocarditis compared with the general population [19]. Besides this, aortic valve replacement has been shown not to prevent the occurrence of lifelong ascending aortic complications [11, 12, 20]. Another aspect recently investigated, and to be regarded in clinical practice, is the association between BAV and cerebrovascular events. Huntley *et al*. [21] analyzed the characteristics of cerebrovascular events in 5401 patients with BAV, from 1975 to 2015, finding that cardioembolism was the leading cause of cerebral or retinal infarction, often from a calcific BAV, and that in 41% of patients, the infarction was recurrent.

## 3. Anatomical Consideration and Classifications

The BAV is featured by the fusion of two contiguous cusps, often with in presence of a raphe of varying degrees and several orientations, which can compromise its proper function. Other anomalies may be the number of aortic sinuses (2 or 3), and a larger and elliptic aortic annulus. In most cases, the fusion of the cusps and the presence of the raphe lead to asymmetry of the orifice. BAV is only symmetric in 5% of cases, with 2 cusps occupying 180° of the annular circumference, without raphe, and only 2 sinuses of Valsalva (“true BAV”) [22]. Most commonly, BAV patients show left coronary artery dominance, shorter left main coronary artery, separate left anterior descending and circumflex ostia, and other coronary artery branch variants [23].

Several classifications of BAV have been released over the years, to characterize and categorize the morphology of the various sub-types [24, 25, 26].

**Fig. 2.**
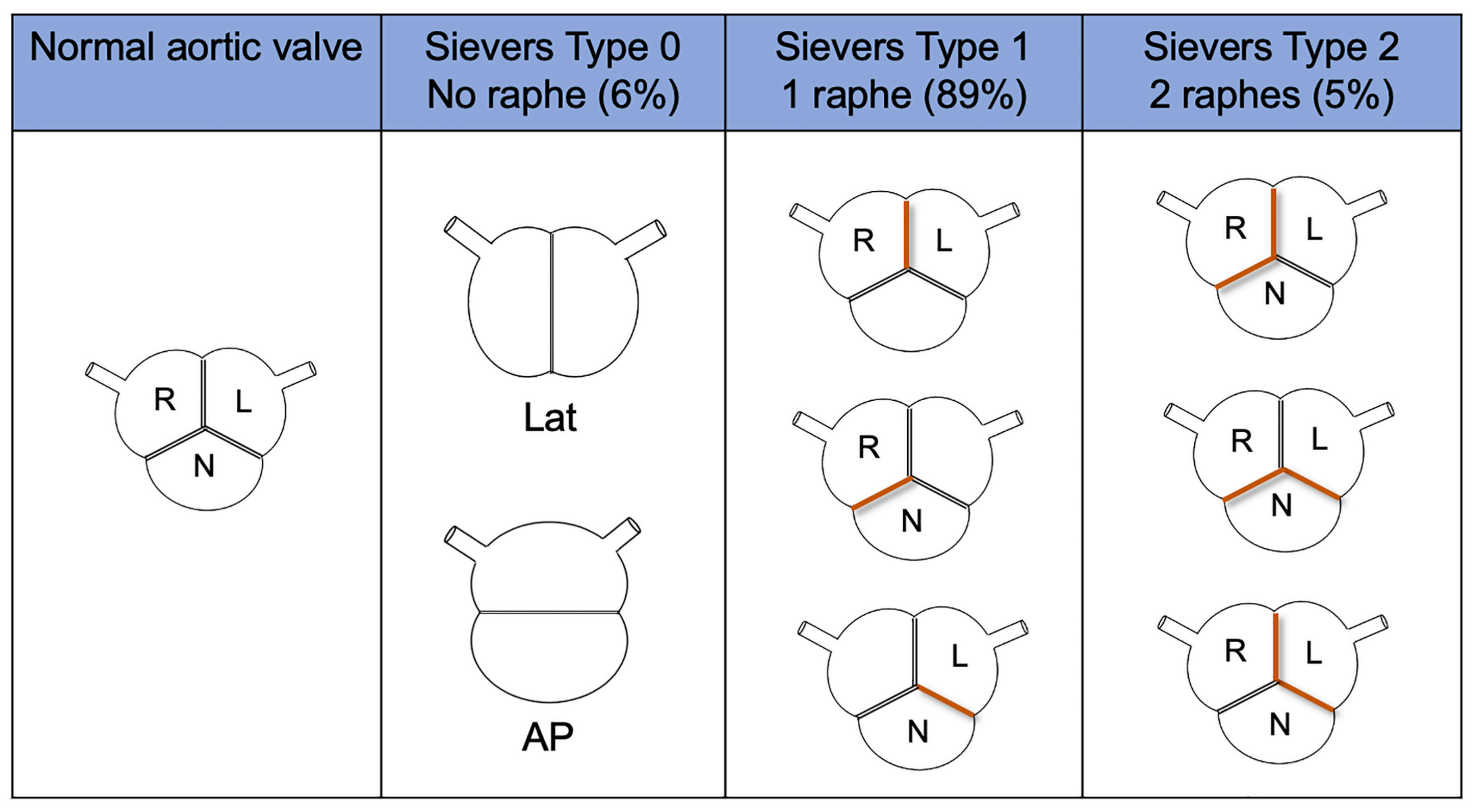
**Sievers and Schmidtke classification system**. AP, antero-posterior; L, left coronary cusp; Lat, lateral; N, non-coronary cusp; R, right coronary cusp.

The Sievers and Schmidtke [24] classification system is one of the earliest and most widely used (Fig. 2). It is based on the number of raphes, that define the type, and the spatial position of the cusps or raphes. Data have been originally taken from operative reports [24]. Type 1 is the most common, with a prevalence ranging from 80–90% in different studies; in particular, the left-right (L-R) coronary raphe subtype is present in 70% of cases and the right-non coronary (R-N) in about 10–20% [11, 22]. Moreover, type 1 L-R has shown a higher incidence of aortic regurgitation compared to type 1 R-N, which has shown a higher incidence of stenosis [27].

In 2021, an international consensus statement on congenital BAV and its aortopathy nomenclature and classification was released by Michelena. It is based on anatomical, clinical, surgical, and pathological data. It is a more comprehensive classification, which includes all BAV and aortopathy phenotypes, using simpler and more descriptive language [28].

**Fig. 3.**
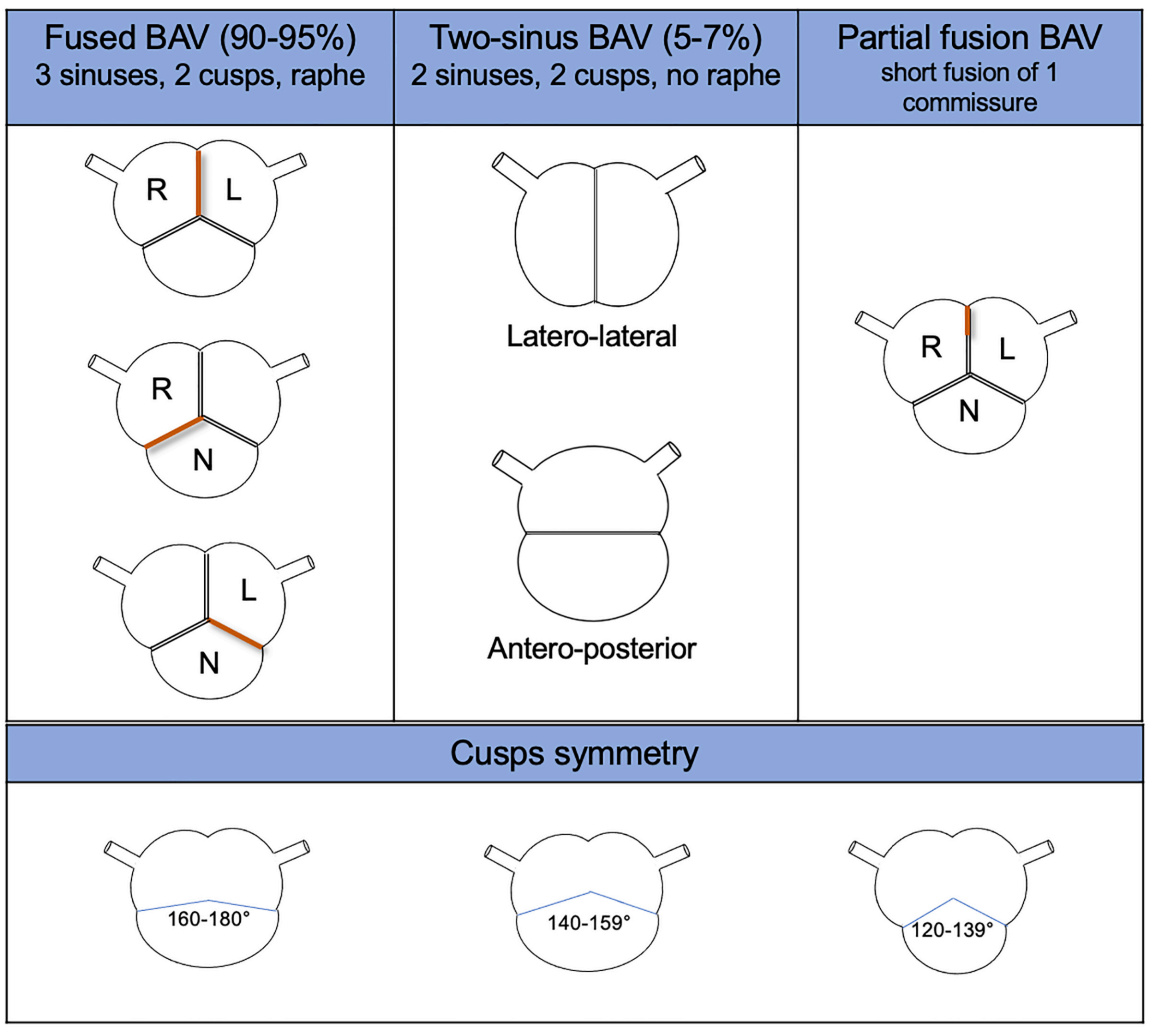
**BAV type, phenotype, and features from the international consensus statement on nomenclature and classification of the congenital BAV**. BAV, bicuspid aortic valve; L, left coronary cusp; N, non-coronary cusp; R, right coronary cusp.

**Fig. 4.**
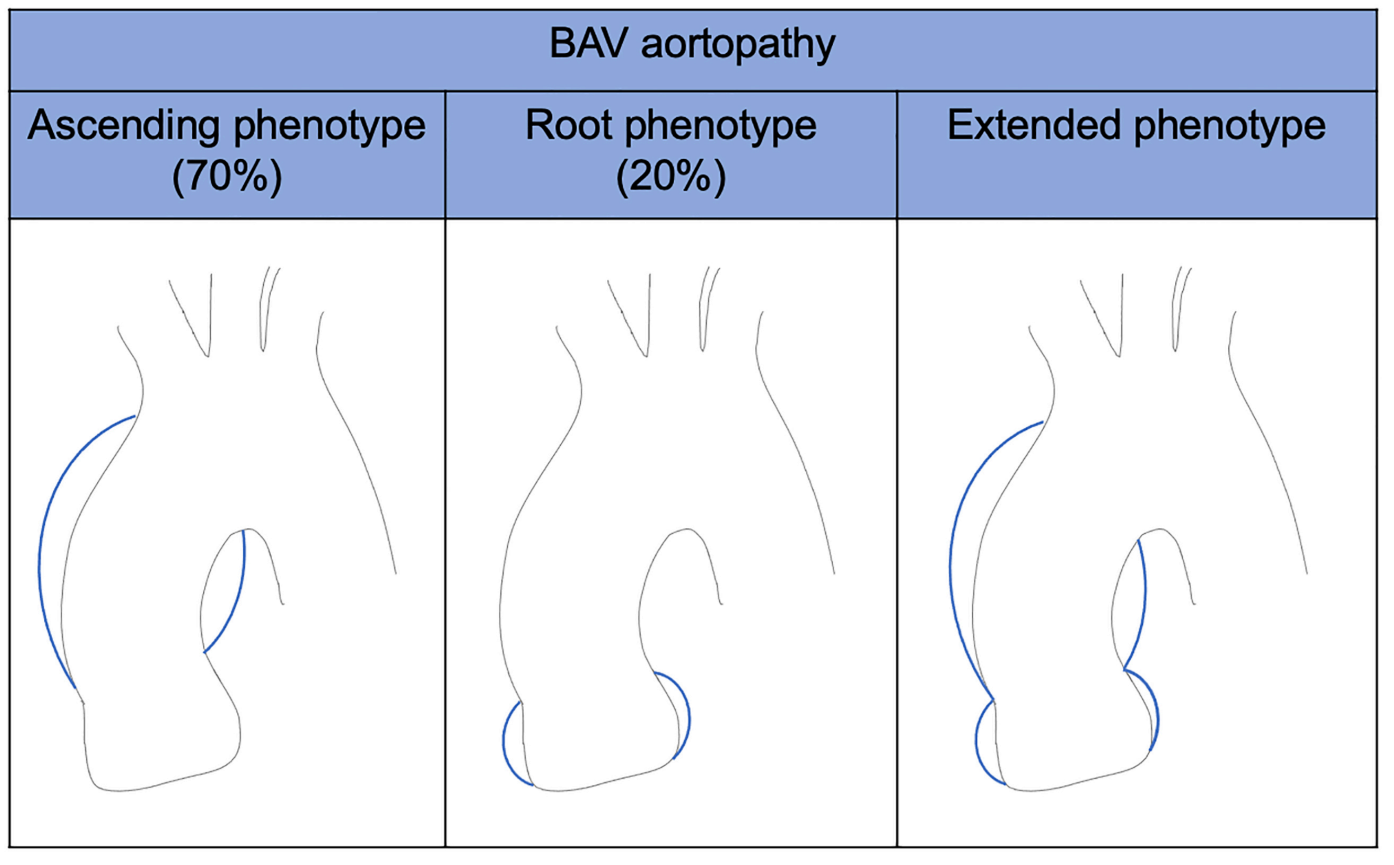
**BAV aortopathy phenotypes**. BAV, bicuspid aortic valve.

They identified 3 BAV types (the fused BAV, the two-sinus BAV, and the partial-fusion BAV), each one with specific phenotypes, based on the presence or absence of raphe and the different possible symmetries of the cusps. The rate of occurrence of each cluster was also described (Fig. 3). Moreover, they identified BAV aorto-phenotypes; among them, the ascending phenotype is common in adult patients with valvular stenosis, while the root phenotype is typical of young patients with valvular regurgitation (Fig. 4). BAV with right-left cusp fusion can be associated with either aortic phenotype.

In their comprehensive analysis, they recognized congenital BAV as a heterogenous valvulo-aortopathological condition in which they identified 3 clinical-prognostic subgroups:

(a) Complex valvulo-aortopathy, typical of young patients requiring early surgery and close surveillance.

(b) Typical valvulo-aortopathy, the most common group, which occurs during adulthood with progressive valve dysfunction or aortic dilatation, requiring long-term surveillance.

(c) Undiagnosed or uncomplicated BAV, which remains silent, but sometimes manifests as an incidental finding.

Yang *et al*. [17], in their retrospective study with long-term follow-up, found that survival of patients with typical valvulo-aortopathy was similar to an age-sex-matched population. Conversely, survival of patients with complex valvulo-aortopathy was lower than expected, with a relative excess mortality risk of 2.25 [17].

## 4. BAV and Rhythm Disorders

Along with the previously mentioned valvular and aortic complications, emerging evidence suggests an increased prevalence of arrhythmias in BAV patients [29, 30]. Among these arrhythmias, atrial fibrillation (AF) and ventricular arrhythmias have pivotal clinical implications.

### 4.1 Atrial Arrhythmias

AF is the most prevalent sustained arrhythmia in patients with BAV. Several mechanisms contribute to the increased susceptibility to AF in this population. Hemodynamic abnormalities, caused by aortic stenosis or regurgitation, can increase left atrial pressure, triggering stretch-induced electrical remodeling [25, 31, 32]. Moreover, structural abnormalities, including aortic root dilation and aortopathy are common in BAV and may generate left atrium electro-anatomical remodeling through inflammation, dilation, and fibrosis, paving the way for AF development [33, 34]. Genetic factors may also play a role, as mutations associated with BAV have been implicated in the pathogenesis of arrhythmias. *NOTCH1*, *GATA4*, and *TBX5 *are the genes with the strongest evidence of this correlation [35, 36, 37].

Albeit, no different treatment is required for AF in BAV patients; a careful screening can be beneficial for an early diagnosis, avoiding symptom misinterpretation.

### 4.2 Ventricular Arrhythmias

Ventricular arrhythmias encompass a spectrum of abnormalities ranging from premature ventricular contractions (PVCs) to life-threatening manifestations such as ventricular tachycardia (VT) and ventricular fibrillation (VF). The presence of ventricular arrhythmias has been linked to an increased risk of sudden cardiac death, requiring careful risk stratification and management [38]. The underlying mechanisms of ventricular arrhythmias in BAV are multifactorial and may include myocardial fibrosis, abnormal myocardial strain due to valvular dysfunction, and genetic predisposition.

PVCs are by far the most frequent arrhythmia in these patients, with an increased prevalence both at rest and during exercise in comparison to normal hearts [29, 30]. Left and right ventricular outflow tracts are the most common origins of PVCs but their burden appears not to be linked to valve disease severity. A possible explanation for this finding is the presence of automatic foci in the outflow tract, caused by the abnormal migration of the neural crest cells in the BAV, more than the development of re-entry circuits due to disease-related hemodynamic changes. The latter, when present, may cause more severe arrhythmias [39]. In fact, monomorphic and polymorphic VT, torsades de pointes (TdP), and bundle branch reentry tachycardia are well reported in patients with BAV, but often in a more advanced and compromised valvular setting [40, 41, 42]. Detection and management of supraventricular and ventricular arrhythmias in patients with BAV are crucial, as their presence significantly increases the risk of stroke, heart failure, and mortality, underscoring the importance of early diagnosis and treatment. Lately, BAV patients undergoing electrophysiology studies have shown a prolonged His-ventricular interval and an increased requirement for a permanent pacemaker [43].

## 5. Diagnosis and Surveillance

**Fig. 5.**
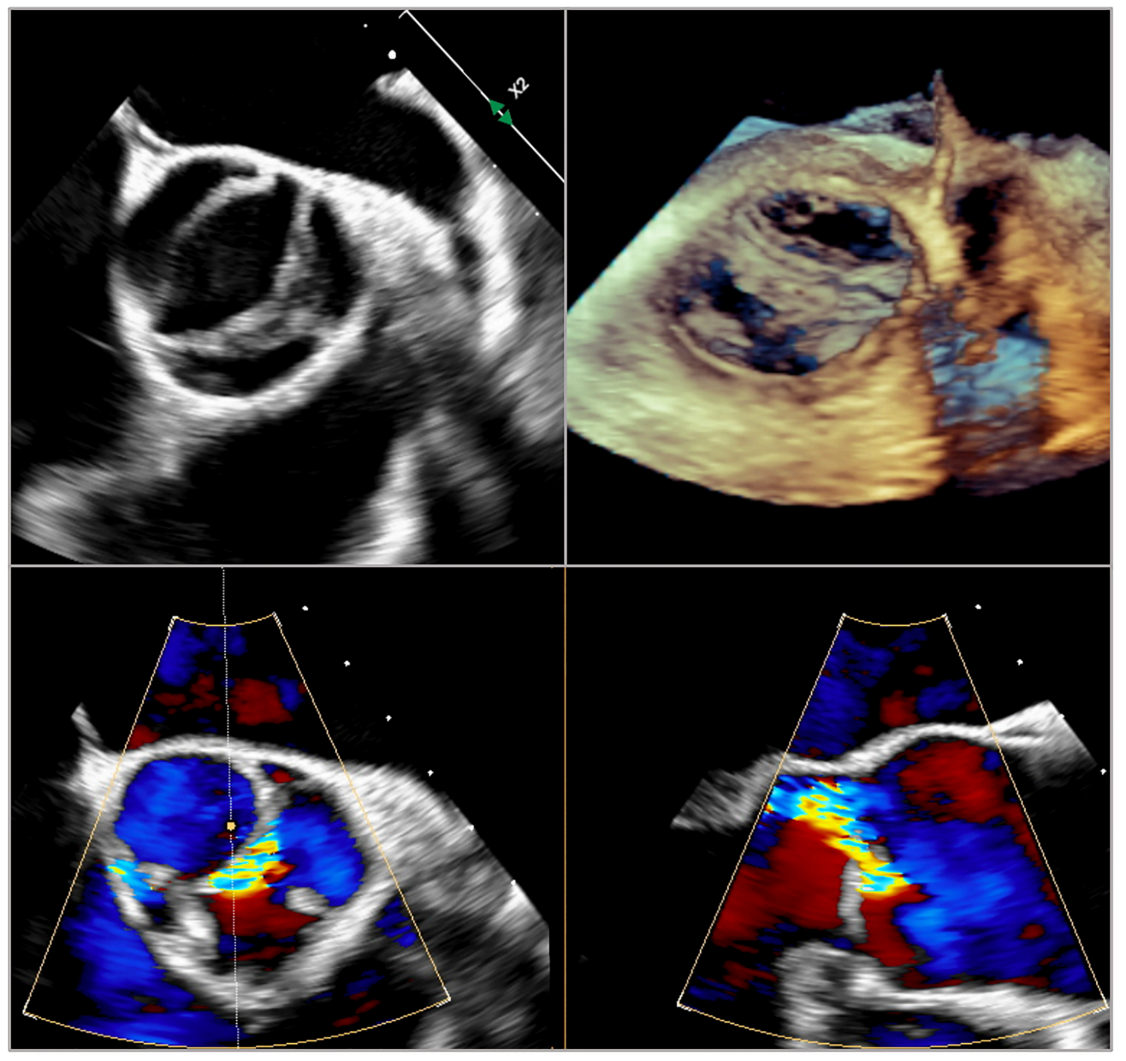
**Transesophageal echocardiographic images of bicuspid aortic valve Siervers type 1 right-left or Fused type right-left**.

The diagnosis of BAV is usually made with transthoracic echocardiography (TTE), which also allows an evaluation of aortic valve phenotype and function, measurement of aortic root and ascending aorta, and assessment of whether there is any associated anomaly. Further imaging with computed tomography (CT) or magnetic resonance imaging (MRI) is recommended for evaluating the ascending aorta, when it cannot be accurately assessed with TTE or when there are coexisting anomalies requiring evaluation (Fig. 5) [44].

BAV patients, with or without aortopathy, require lifelong surveillance. Indeed, the progression of valve disease and growth of the aorta can occur in the absence of symptoms.

The 2020 American College of Cardiology/America Heart Association Guideline for the ‘Management of Patients With Valvular Heart Disease’ suggested periodic imaging, with TTE, CT, or MRI, in adults with BAV and sinuses or ascending aorta ≥40 mm, with an interval determined by the rate of progression of the valvulo-aortopathy and associated risk factors, like family history of dissection. Even BAV patients who previously underwent aortic valve replacement should continue lifelong periodic imaging, given their increased risk of aortic dilatation and dissection [44].

Further, the framing of rhythm disorders in patients with BAV is of emerging importance. Holter Electrocardiographic monitoring and careful symptom assessment are pivotal for comprehensive BAV management.

Genetic testing of individuals with BAV and their relatives is not recommended, given the complex inheritance and the lack, to date, of genes with a defined prognostic significance.

Screening of first-degree relatives’ aortic valve and ascending aorta with TTE should be considered, in light of the high prevalence of family association [44]. However, the actual cost-effectiveness and clinical implications are still to be investigated and remain a matter of debate [7].

Currently, novel biomarkers have been identified with a causal association with aortic stenosis. In particular matrix metalloproteinase 12 (MMP12) and complement C1q tumor necrosis factor–related protein 1 (C1QTNF1) were associated with a greater increase in aortic valve peak velocity and a greater degree of aortic valve calcifications [45]. These findings may allow early identification of patients with BAV at higher risk of valve dysfunction.

## 6. BAV Surgical Management

Surgery remains the gold standard in patients with acceptable surgical risk and in cases of associated aortopathy [46]. When it comes to surgical treatment, however, special attention should be paid to evaluating the opportunity to perform aortic valve repair. While BAV-associated aortic stenosis usually develops during the 6th–7th decade of a patient’s life, aortic regurgitation affects younger people, and it is still a matter of debate whether a mechanical prosthesis (MP) represents the best option for the lifetime management of these subjects [47, 48, 49].

### 6.1 Isolated BAV Disease

Both European and American guidelines recommend performing aortic valve replacement (AVR) in patients with severe aortic valve disease (either stenosis or regurgitation) if symptomatic or, in the absence of clear symptoms, if either left ventricular dysfunction or another indication for cardiac surgery exists. These recommendations are applicable irrespective of aortic valve morphology [44, 46].

Special attention should be paid when dealing with prosthesis choice, considering that BAV patients, especially those with severe aortic regurgitation, are typically young.

In fact, even though MP is more durable than biological ones, the implantation of an MP significantly affects the long-term survival of non-elderly subjects, with a mortality rate of around 1% per year, higher than the age-matched population [50, 51]. Moreover, long-term anticoagulation impairs the quality of life of these patients, being associated with an increased risk of thrombotic or hemorrhagic phenomena, and it is not a valuable alternative for young girls who wish to become pregnant [52, 53].

For these reasons, different solutions have been investigated over the years to offer young BAV patients an alternative to MP.

#### 6.1.1 Lower the Threshold for Biological Prostheses

European guidelines consider 60 years of age as the cut-off for biological prosthesis (BP) implantation in the aortic position. While American ones are more aggressive on this topic (BP suggested in patients older than 50), both guidelines would suggest the implantation of an MP in young patients with BAV having a surgical option [44, 46].

A trend in lowering this threshold has been recently developed, along with the diffusion of transcatheter alternatives for reintervention in case of BP dysfunction [54].

#### 6.1.2 Ross Procedure

A renewed interest in the Ross procedure (pulmonary autograft replacement) in adults has been recently noted.

Several studies conducted in high-volume centers have shown excellent outcomes, with a long-term survival similar to age- and sex-matched populations and a higher cardiovascular event-free probability if compared with biological or mechanical AVR [55, 56, 57].

Moreover, the Ross procedure is associated with a very low rate of valve-related complications (autograft endocarditis and thromboembolism, bleeding, or valve thrombosis occurring in 0.26% and 0.36% patients/year, respectively) [58].

However, to obtain these good long-term results, patients selection is the key. The main predictors of late failure of the pulmonic autograft include dilated aortic annuli (more than 27 mm), pure aortic regurgitation, and aortic/pulmonary size mismatch [59]. Moreover, tight blood pressure control is imperative after the intervention, in order to avoid pulmonary autograft dilatation.

Possible drawbacks of this intervention are technical complexity, potential long-term failure of both homograft and pulmonic valves, and the difficulty of reoperations, if any [55].

As a matter of fact, in expert hands, the Ross procedure still represents a valid alternative to mechanical AVR in young BAV patients, being linked with a risk of reintervention of only 1–1.5% per patient-year [60].

#### 6.1.3 Aortic Valve Repair

In the last few decades, aortic valve repair, even in BAV, has become a possible alternative to AVR, capable of ensuring optimal survival and a durability of up to 20 years, with the recommendation that it be performed in selected patients and experienced centers [48, 61].

Over time, various predictors of the long-term success of the repair have been described, namely effective height, geometric height, annular dilatation, and commissural orientation.

- The effective height (EH) is the distance between the central free margin of the cusp and the annular plane in diastole. An EH of at least 9 mm after the repair has been described as a predictor of long-term durability. In fact, such a cut-off helps to minimize the risk of undetected symmetric prolapse as a mechanism of valve repair failure [62, 63]. EH may be assessed either through pre-and post-operative echocardiography or intraoperatively, using dedicated calipers.
- The geometric height (GH) is the distance between the free margin and the nadir of the cusp and represents an indicator of the amount of cusp tissue available for repair. A GH <18 mm has been considered as an indicator of cusp retraction and, consequently, a predictor of poor long-term results in cases of valve repair [64, 65].
- Annular dilatation is present in most of the patients with pure aortic regurgitation. An annular diameter >25–27 mm was found to be an independent risk factor for repair failure [66]. Consequently, different annuloplasty techniques have been developed (subcommissural plication, suture annuloplasty, external ring implantation), with a substantial improvement in repair durability [67]. However, aortic annuloplasty is technically demanding, as a deep dissection has to be made to reach the virtual basal ring and careful attention is needed to avoid injury to the left coronary artery.
- Commissural orientation: a commissural angle between 160 and 180° has been associated with the best hemodynamic (lower systolic gradients) and durability of BAV repair [24]. In the case of angles <160°, plication of aortic walls or aortic root replacement (if dilated Valsalva sinuses) have been proposed as techniques to achieve a good commissural orientation.

Anyhow, highly retracted leaflets or a severely calcified raphe may represent contraindications to BAV repair. Even though pericardial patch reconstruction is feasible, the use of the pericardium as partial cusp replacement has been described as a predictor of repair failure, especially in BAV patients [68].

Recent studies, comparing the results of the aortic valve-sparing surgery between BAV and the tricuspid aortic valve, demonstrated a similar rate of reintervention within 4 years, after that BAV patients had an increased incidence of reintervention [69].

#### 6.1.4 Ozaki Procedure

Another interesting alternative to AVR with an MP is the aortic valve neocuspidization using glutaraldehyde-treated autologous pericardium, called Ozaki procedure after his inventor.

This technique was first described in 2007 and, since then, has been associated with good mid-term outcomes (freedom from more than moderate aortic regurgitation 97.6 ± 1.7% at 5 years, 92.7% at 9.8 years), also in patients with BAV [70, 71]. Patients with dilated aortic sinuses (>45 mm) or aortic annulus (>29 mm) are not good candidates for this technique, as it does not fix either annular or sinus dimensions [62].

Anyhow, long-term results are still missing and consequently, a word of caution should be spent before adopting this technique in very young patients.

### 6.2 BAV Disease with Concomitant Aortopathy

Almost 50% of individuals with BAV develop an ascending aortic aneurysm during their lifetime and may need surgery to prevent aortic complications [48].

The most common variant of aortopathy in BAV patients is an ascending aorta aneurysm, with varying degrees of root dilatation. Isolated root dilatation is rare and commonly associated with aortic regurgitation in young patients [72].

In subjects with BAV, international guidelines recommend ascending aorta/aortic root replacement if the aortic diameter is more than 55 mm. In the presence of additional risk factors (i.e., family history of aortic dissection, uncontrolled arterial hypertension, aortic size increase >3 mm/year), surgery may be considered when aortic size exceeds 50 mm. Moreover, if aortic valve disease is triggering surgery, aortic replacement is reasonable when the aortic diameter is more than 45 mm [44, 46]. Special attention should be paid, however, when performing aortic valve repair. Indeed, root replacement has been associated with improved repair durability, even in mildly dilated aortas, thanks to the possibility of obtaining a good commissural orientation [48, 66].

On the other hand, whether to perform AVR in patients with moderate BAV disease undergoing ascending aorta replacement is still a matter of debate. Frequently the progression of valvulopathy in these patients is slow, and only 5% of them would eventually develop severe aortic regurgitation/aortic stenosis at 12 years follow-up [73]. A patient-tailored approach is needed in this context, also considering the patient’s age and wishes [73].

## 7. BAV Transcatheter Management

The first human implantation of a transcatheter aortic valve (transcatether aortic valve replacement, TAVR) was performed by Alain Cribier [74], in 2002, in a “last-resort” patient with a severely calcified BAV and early series with first-generation TAVR devices in BAV patients showed safety and feasibility [75].

Despite these findings, BAV was initially considered an exclusion criterion for TAVR due to anatomical and procedural factors which made the implantation more challenging [76].

The predominantly elliptical rather than spherical shape of the orifice, usually encountered with BAV, and the frequently associated ascending aorta dilatation and weakness, make placement and attachment of a transcatheter valve more difficult and at risk of aortic injury [77, 78]. In addition, valve asymmetries due to raphe and different calcification patterns could contribute to valve misalignment or under-expansion during valve deployment. All these elements may lead to a higher risk of paravalvular leaks (PVL), early valve deterioration, and an increased need for pacemaker implantation [12, 79]. Increased understanding of valve morphology, sizing techniques and the introduction of new-generation transcatheter valves, with design improvement and better sealing methods, have made it possible to overcome some of these issues [80]. However, considering the early onset of stenosis in BAV patients, the excellent results of the surgery, and the lack of studies investigating the outcomes of TAVR in different BAV subtypes, currently the guidelines consider TAVR as an alternative to surgical aortic valve replacement (SAVR) “after consideration of patient-specific procedural risks, values, trade-offs, and preferences, and when the surgery is performed at a Comprehensive Valve Center” [44, 77, 81].

### 7.1 Sizing Methods 

From general practice and literature with TAVR in tricuspid valves, it is known that pre-procedural planning is essential to obtain the correct positioning and to minimize any complications. Due to BAV’s peculiar characteristics, a TAVR-specific classification was recently introduced by Jilaihawi *et al*. [82] to categorize morphological anomalies that influence the interaction between TAVR and BAV. These authors distinguished three types of BAV according to the number of commissures and the presence of raphe:

(a) tricommissural or acquired BAV, arising from the degenerative process of a tricuspid aortic valve;

(b) bicommissural raphe-type (Sievers type 1);

(c) bicommissural non-raphe-type (Sievers type 0).

The presence of raphe and its degree of calcification has important implications regarding adequate prosthesis expansion.

Multislice computed tomography (MSCT) is the gold standard for annular sizing, characterization of valve morphologies, determination of risk of complications (e.g., annular injury, coronary occlusion, etc.), assessment of the ascending aorta, and vascular access.

Tarantini and Fabris recently published a practical overview of periprocedural operative consideration for TAVR in this context [83].

In BAV, two planes should be sought during systole: the annulus plane and the supra-annular one.

The annulus plane or virtual basal ring is the same used for tricuspid valves, and it represents the correct plane for prosthesis selection, sizing, and implantation height. It is identified as connecting the hinge points of the basal attachments of the aortic valve cusps; in BAV this may be more challenging due to fused cusps or their unequal size.

**Fig. 6.**
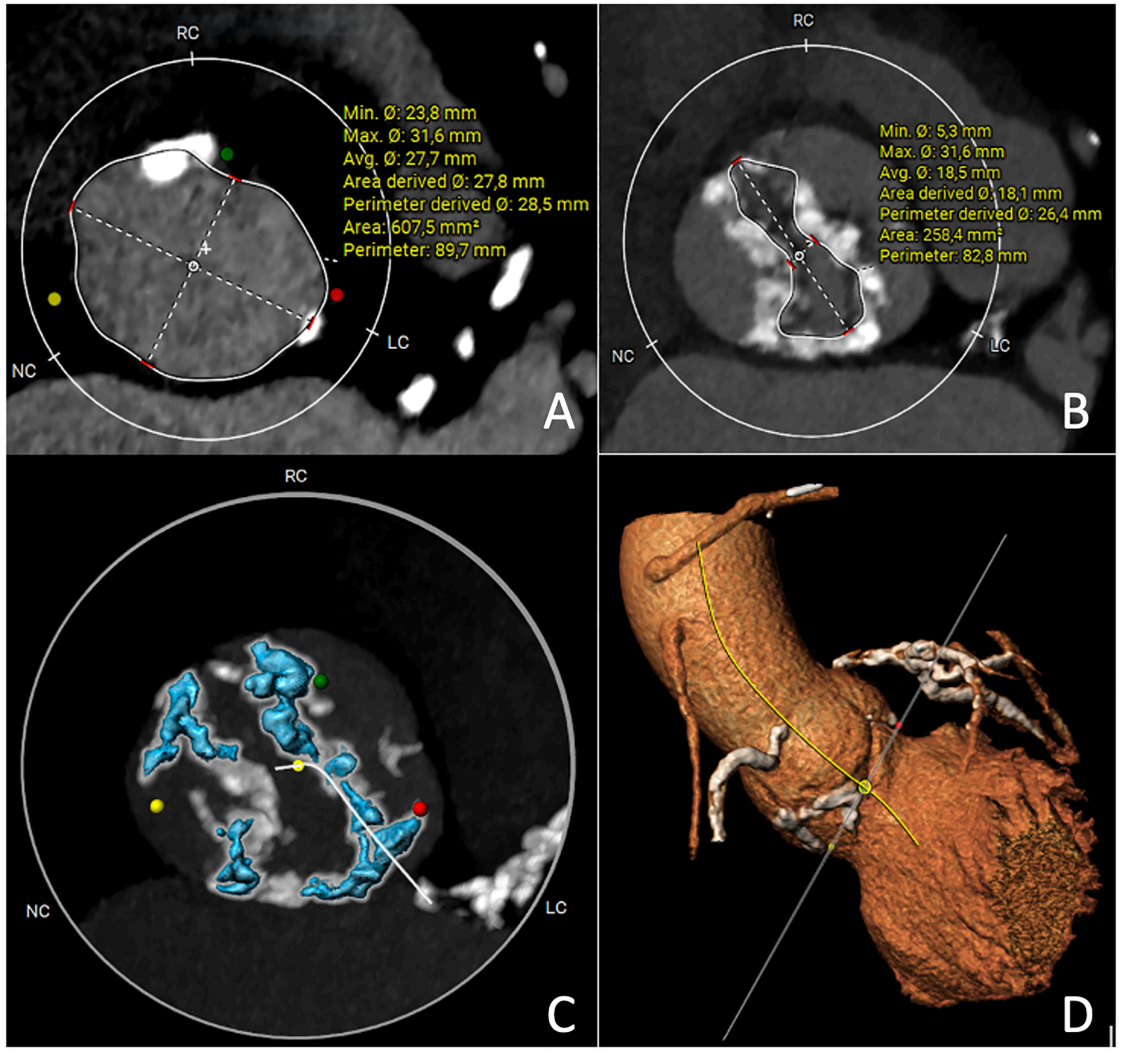
**Multislice computed tomography images of bicuspid aortic valve Sievers 1 right-left or Fused type right-left**. (A) Virtual Basal Ring measurement. (B) Virtual Raphe Ring measurement. (C) Calcium quantification HU 900. (D) Three cusps aorta image. HU, Hounsfield unit; RC, right coronary cusp; NC, non-coronary cusp; LC, left coronary cusp.

The supra-annular plane or virtual raphe ring may be the narrowest part of the aortic root in the BAV, and in this case the anchoring point of the prosthesis, its measurement can help in procedure planning and predicting implant outcome (Fig. 6). At this level, it is possible to identify inter-commissural distance and perform raphe-specific evaluation. Overall, many supra-annular sizing methods exist, with no consistent recommendation on which height to measure and no consistent tools or techniques on how to measure it.

In addition, intra-procedural balloon-sizing and valvuloplasty allow for assessment of the size of the prosthesis, to facilitate valve positioning, to evaluate raphe stiffness and possible coronary obstruction. Balloon post-dilatation could be a way to address prosthesis distortion in case of PVL or transvalvular gradient, taking care of possibly serious complications [84].

Yoon *et al*. [85] in a series of 108 patients treated with balloon-expandable devices reported a rate of 0.9% for annulus rupture and 6.5% for more than moderate PVL. In the experience of Mylotte *et al*. [86], balloon-expandable valves had a rate of annulus rupture of 0.7%, whereas a more than moderate final PVL was reported in 6% of patients. In the perspective of a multi-parametric evaluation, two algorithms were proposed. The Casper algorithm is based on raphe extension and calcium distribution [87]. The Lira method aims to recognize the plane where valve anchoring is assumed [88]. Several registries are investigating the results of different sizing methods (BIVOLUTX NCT03495050 and CASPER registry NCT04817735).

### 7.2 Prosthesis Selection: Self-expandable versus Balloon-expandable

To date, there is no definitive recommendation on the type of transcatheter prosthesis to be used (balloon-expandable vs self-expandable), in patients with BAV.

In the BEAT (balloon versus self-expandable valve for the treatment of BAV stenosis) registry, which included 353 consecutive patients who underwent TAVR using new-generation Evolut R/PRO or Sapien 3 valves [89], the Valve Academic Research Consortium-2 device success was similar between both groups. After propensity score matching, there were no differences in the rate of permanent pacemaker implants and at 1-year follow-up, the rate of overall death and cardiovascular death were similar. However, the balloon-expandable Sapien 3 valve (Edwards Lifesciences) showed a higher rate of annular rupture while the self-expanding CoreValve Evolut R/PRO valve (Medtronic) had higher rates of PVL.

In a meta-analysis of eight observational studies from 2013 to 2020 including 1080 patients with BAV stenosis (620 treated with a balloon-expandable valve and 460 with a self-expanding one), the balloon-expandable valves showed a statistically significant higher risk of annulus rupture, while the new generation balloon-expandable ones were associated with significantly less paravalvular leak when compared to the new generation of self-expanding valves [90].

Finally in the most recent TRITON study, a multicenter registry of 360 consecutive patients with severe BAV stenosis treated with balloon-expandable transcatheter valves (Myval and SAPIEN 3 Ultra) or self-expanding Evolut PRO+, the authors found that, at 30 days, all valves showed similar safety but both balloon-expandable devices had lower residual aortic regurgitation due to PVL than the Evolut PRO+ [91].

### 7.3 Complications, Challenges, and Future Directions 

PVL and the necessity of pacemaker implantation are the major concerns regarding TAVR in BAV patients. The burden and distribution of calcification remain an independent risk factor for mortality, root injury and PVL, as shown in Yoon’s study [92] (1034 BAV patients from the International BAV stenosis Registry, treated with Sapien3 or EvolutR/Pro valves). This factor is also strongly associated with the increased risk of stroke observed in BAV compared with tricuspid aortic valve patients undergoing TAVR [93]. Although the rate of pacemaker implantation has decreased since the first studies, to date it remains consistently high. In a low-risk trial comparing TAVR in trileaflet and bileaflet stenosis, at 2-year follow-up there was no significant difference in mortality, stroke and PVL in bicuspid versus tricuspid patients, but 16% of BAV patients required permanent pacemaker implantation compared to 8.6% of tricuspid ones [94].

In the future, the introduction of non-implant-based devices, like the Leaflex device (Pi-Cardia, Rehovot, Israel, a transcatheter solution that modifies leaflets calcification to increase mobility and improve the function of the valve) will probably be able to overcome the limits described above. A first-in-human study was performed in sixteen patients subsequently treated with TAVR and demonstrated its safety, feasibility and performance [95].

Despite the excellent results reported even in low-risk patients, there are still a lack of trials directly comparing the results of surgery with the transcatheter treatment of BAV patients, of which we know only short-term results. Further, patients with BAV often have premature valve degeneration associated with early aortic stenosis. This finding, in addition to the frequently associated aortopathy, means that many patients with BAV, after TAVR, would frequently require surgery combined with ascending aorta treatment.

It should also not be forgotten that a significant percentage of patients with BAV may present with isolated aortic regurgitation, for which TAVR has shown a high rate of embolization and risk of PVL. Two dedicated transcatheter valves for aortic regurgitation are the J-Valve (JC Medical) and the JenaValve (JenaValve Technology) whose technology enables alignment and anchorage to the native valve leaflets. Preliminary data appear promising particularly in tricuspid aortic valves [96].

## 8. Conclusions

In recent decades, much has been done to understand the genetics, anatomy, and aetiopathogenesis of BAV and BAV-related aortopathy. The advancements in the diagnostic and imaging modalities have allowed a more detailed description and understanding of BAV and helped to introduce new nomenclature, classification, and categorization of BAV phenotypes. Despite this, much remains to be done on the prevention and prediction of the development of clinical complications. The techniques of diagnosis and surgical treatment, refined over time, have allowed for ever better outcomes, with the advent of transcatheter techniques increasing the possibility of minimally invasive treatments foe inoperable or high-risk patients with a view to an increasingly patient-specific approach.
